# Evaluation of genome scaffolding tools using pooled clone sequencing

**DOI:** 10.3906/biy-1805-42

**Published:** 2018-12-10

**Authors:** Elif DAL, Can ALKAN

**Affiliations:** 1 Department of Computer Engineering, Faculty of Engineering, Bilkent University , Ankara , Turkey

**Keywords:** Genome assembly and scaffolding, high-throughput sequencing, pooled clone sequencing, systems biology

## Abstract

DNA sequencing technologies hold great promise in generating information that will guide scientists to understand how the genome effects human health and organismal evolution. The process of generating raw genome sequence data becomes cheaper and faster, but more error-prone. Assembly of such data into high-quality finished genome sequences remains challenging. Many genome assembly tools are available, but they differ in terms of their performance and their final output. More importantly, it remains largely unclear how to best assess the quality of assembled genome sequences. Here we evaluate the accuracies of several genome scaffolding algorithms using two different types of data generated from the genome of the same human individual: whole genome shotgun sequencing (WGS) and pooled clone sequencing (PCS). We observe that it is possible to obtain better assemblies if PCS data are used, compared to using only WGS data. However, the current scaffolding algorithms are developed only for WGS, and PCS-aware scaffolding algorithms remain an open problem.

## 1. Introduction

Completion of the Human Genome Project (HGP) was one of the greatest achievements in all life sciences research (International Human Genome Sequencing Consortium, 2004). The HGP was started in 1990, and thanks to the innovations in automated genome sequencing technologies, the human genome was completed in 2004. Today, >97% of the human genome is finished and released as the human reference genome (version GRCh38). The HGP has allowed researchers to learn functions of genes and effects of their mutations, and it was the driving force and motivation for the 1000 Genomes Project (The 1000 Genomes Project Consortium, 2015). The information we gain thanks to the reference genome built by the HGP and the subsequent analyses performed by the 1000 Genomes Project and the ENCODE Project (ENCODE Project Consortium, 2012) will be the main source of knowledge in achieving precision medicine.

The first genome assembly algorithms were designed in the early 1980s and 1990s (Kececioglu and Myers, 1995) ,followed by the development of many different assemblers that make use of different methodologies (Sutton et al., 1995; Batzoglou et al., 2002; Mullikin and Ning, 2003). With the help of emerging technologies, more powerful computers, and massively parallel high-throughput sequencing (HTS), scientists are now able to read and assemble genomes faster than ever before (Mardis, 2008).

The assembly process is much like assembling a jigsaw puzzle, trying to find the original places of each puzzle piece by checking each piece next to each other to see if they fit together. Computationally, it is similar to the shortest superstring problem, known to be NP-complete, where approximation algorithms still need to perform billions of sufix-to-prefix comparisons, or extract and compare all k-mers, even when short sequences are assumed to be error-free (Steinberg et al., 2017) . When sequencing errors are considered in genome assembly, each piece of a DNA fragment is sequenced several times to correct for the errors, making the computational burden more pronounced.

Creating a digital representation of a genome is achieved in three main steps. First, the genome (collection of chromosomes) is fragmented into shorter pieces, then sequenced using HTS technologies (Mardis, 2008; Shendure and Ji, 2008). Second, the billions of short reads are evaluated to be assembled together to reconstruct the original genome sequence using either prefix-sufix overlaps (Batzoglou et al., 2002; Simpson and Durbin, 2012) or de Bruijn graphs (Chaisson et al., 2004; Zerbino and Birney, 2008; Simpson et al., 2009). In this step, contiguous segments (termed contigs) are obtained. Contigs are long sequences without any information about their order and orientation in the genome. To enhance the assembly to include relative order and orientation of these contigs, scaffolding algorithms are used (Steinberg et al., 2017). Scaoflding is briefly defined as delineating the order and orientation of the contigs through “linking” them together by estimating the gaps between contigs.

Efforts of assembling large and complex genomes, such as human (International Human Genome Sequencing Consortium, 2004) , gorilla (Scally et al., 2012) , pine (Zimin et al., 2014) , and others, always resulted in assemblies fragmented into variably sized hundreds of thousands of contigs. This is because of several factors: the complexity of the genome (i.e. repeats and duplication content), errors imposed by the sequencing methodology, and depth of sequencing coverage. The human reference genome is largely constructed using the Sanger sequencing technology, in a hierarchical manner using BAC and plasmid cloning vectors. Sanger technology is able to generate long reads (700–1000 base pairs) to be sequenced with a very low error rate (International Human Genome Sequencing Consortium, 2004). However, it is also very costly: the HGP cost over 3 billion US dollars to complete. Newer sequencing technologies, commonly referred to as HTS, were first realized in 2005 (Margulies et al., 2005) and have evolved very rapidly since then. Although the most widely used HTS technology (i.e. Illumina) produces short reads (100–150 base pairs) with a higher error rate (~0.1%), the associated costs are substantially less, and it is possible to generate billions of reads in a single run. This enables these technologies to provide data at high redundancy, measured as depth of coverage, which in turn makes it possible to ameliorate the effect of sequencing errors.

The most difficult problem in genome assembly seems to be resolving repeats and ensuring comprehensiveness (Treangen and Salzberg, 2012). A relatively new technology, called pooled clone sequencing (Kitzman et al., 2011), aims to merge the cost efficiency of whole genome shotgun sequencing (WGS) with the repeatresolving abilities of clone-based hierarchical sequencing, which was employed by HGP. A newer version of the same strategy is the recently announced linked-read sequencing method by the 10x Genomics company (Mostovoy et al., 2016).

In this paper we evaluate the efficacy of various genome scaffolding algorithms when pooled clone sequencing data are available and compare them against assemblies generated with WGS-only data. Here we benchmark four different scaffolding tools: Opera (Gao et al., 2011), SCARPA (Donmez and Brudno, 2013), SSPACE (Boetzer et al., 2011), and BESST (Sahlin et al., 2014), where we assemble the longest and the shortest human chromosomes (1 and 20) and compare them with the assembly generated with ALLPATHS assembler (Gnerre et al., 2011). The pooled clone sequencing dataset that we use in this study was generated from the genome of the same individual with the WGS data (NA12878), divided into 288 separate pools that were sequenced using the Illumina technology (Kitzman et al., 2011). In this manuscript we do not focus on computational requirements of different scaffolding algorithms, and we recommend another publication (Hunt et al., 2014) to the interested reader.

## 2. Materials and methods

The WGS strategy using HTS is relatively inexpensive but not powerful in resolving repeats, and the clonebased hierarchical sequencing strategy is better for repeat resolution but prohibitively expensive. Therefore, to leverage the strengths of both strategies, we propose to use a hybrid approach named pooled clone sequencing (PCS) originally developed for haplotype phasing (Kitzman et al.,2011).

## 2.1. Pooled clone sequencing

In this work, we used the genome of NA12878, an individual from Utah of North European ancestry. We obtained the data from the lab of Evan Eichler from the University of Washington, and this dataset was previously published in a study to characterize genomic structural variation (Eslami Rasekh et al., 2017).

First, genomic DNA is broken into fragments using restriction enzymes and all diploid fragments are sizeselected using gel electrophoresis. Those fragments with size 150–200 kbp are then cloned using bacterial artificial chromosome (BAC) cloning vectors. After a dense solution of BAC clones are obtained, they are diluted into 288 pools. The main purpose of partitioning the genome into a large number of pools is to prevent overlapping regions from being in the same pool, thus reducing the probability of generating reads from different copies of interspersed genomic repeats in the same sequencing run. In this experiment, each pool contains about 300 BAC clones, which makes it very unlikely that two clones that originate from the same genomic segment are included in the same pool (Kitzman et al., 2011) . Finally, each pool is tagged with sequencing barcodes and sequenced using the Illumina platform at 3–4× depth of coverage. The Figure summarizes the entire protocol. To evaluate the efficacy of PCS in genome scaffolding, we focused on chromosomes 1 and 20 of the human genome, which are the longest and the shortest chromosomes in the latest human reference genome, respectively (GRCh38).

**Figure:**
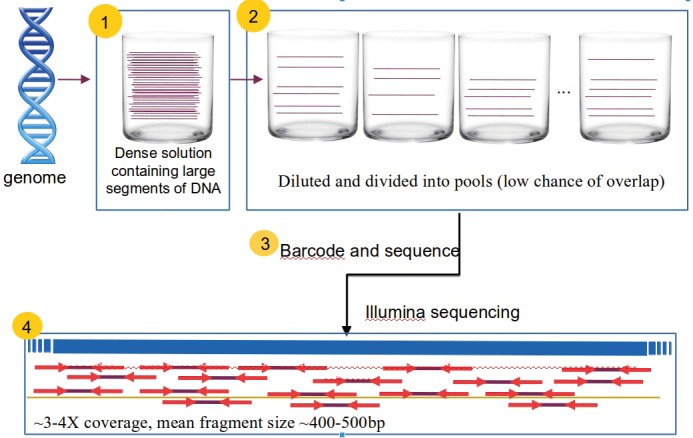
Pooled clone sequencing. 1) A dense solution that contains large segments of DNA is prepared. 2) The collection of genomic fragments is diluted and separated into a large number of pools, resulting in a low chance of overlaps within a pool. 3) DNA in each pool is further fragmented to prepare sequencing libraries and barcodes are attached to be able to separate reads after sequencing. 4) All pools tagged with different barcodes are merged and sequenced using the Illumina platform.

## 2.2. Scaoflding tools used in this study

SSPACE (Boetzer et al., 2011) is the first scaoflder that use reads generated with HTS platforms. Since the scaffolding problem is NP-hard (Gao et al., 2011), the solutions are typically based on heuristics. SSPACE applies a greedy procedure, and it tries to solve the problem by starting with the largest contig first. It maps paired-end reads to contigs and looks for such read pairs that “link” different contigs. After contigs are linked using paired end reads, scaffolds are constructed iteratively by linking contigs if they have a sufficient number of connections between each other. SSPACE requires the minimum number of pairedend reads that connect two contigs to be 5. The distance between contigs is estimated using the insert sizes of the paired-end reads. Ambiguities caused by alternative links are resolved using a threshold on read pair counts, and the scaffolding process continues until no more contigs joined. If no further contig is found to extend the current scaffold, the current scaffold is finalized. The process continues until all contigs are incorporated into scaffolds.

SCARPA (Donmez and Brudno, 2013) uses linear programming to find near-optimal scaffolds. The most challenging problem for scaffolds is misassemblies, and SCARPA tries to fix assembly mistakes during the scaffolding process. As a preprocessing step, SCARPA filters mapping files to remove ambiguous mappings to perform several calculations on the mapping properties, such as the average fragment size and standard deviation. During preprocessing, if SCARPA detects an ambiguity in paired-end read span (i.e. fragment size not within 3 standard deviations of the average length), SCARPA considers that the relevant contig is misassembled and discards it. This increases scaffold accuracy, but also causes loss of data.

Opera (Gao et al., 2011) aims to find an exact solution for scaffolding instead of applying heuristics. Since the scaffolding problem is NP-hard (Gao et al., 2011) , the exact solution cannot be calculated efficiently without any constraints. Therefore, Opera calculates an optimal solution under specified constraints. Opera is a graphbased algorithm, where contigs are represented as nodes and paired end reads that map to contigs form the edges. Initially, two orientations (i.e. strands) are assigned for each contig, and then one orientation is determined using the mapping properties of the paired-end reads. Gao et al. proved that the scaffolding problem cannot be efficiently solved using a scaffold graph without any constraints (Gao et al., 2011). To relax the problem and make it feasible to solve, Opera introduces a lower bound for initial contig lengths and an upper bound for the number of paired-end reads that link the contigs.

BESST is a scaffolding algorithm that differs from others in estimating gap lengths in scaffolds (Sahlin et al., 2014). BESST models the distribution of reads that span a gap and derives a machine learning-based formulation that was previously used by other scaffolds to estimate the gap sizes.

## 3. Results

We evaluated the performance of scaffolding experiments and the efficacy of using PCS data. In the experiments, we used the de novo assembly of the NA12878 genome (Gnerre et al., 2011) as the main contig source and PCS data generated from the same genome (Kitzman et al., 2011; Eslami Rasekh et al., 2017) for scaffolding. To understand the additional benefit of having PCS data, we also merged all reads in the PCS dataset to emulate WGS-based scaffolding (i.e. no additional information from PCS). We investigated the value of the PCS dataset by collectively and hierarchically applying scaffolding pool-by-pool. Collective application of scaffolding refers to the usage of all reads generated in the sequencing experiment, thus discarding the additional information that can be gained from PCS sequencing. On the other hand, hierarchical application of scaffolding refers to running the scaffolding tools for each “pool” of the PCS-generated data, one-byone, in a hierarchical manner. The hierarchical application of scaffolding substantially reduces the probability that reads that may originate from different copies of the same repeat type are handled separately.

## 3.1. Evaluation criteria

We compared the scaffolding performance using four metrics: 1. Number of scaffolds: a lower number of scaffolds is deemed to be better in comparison. An ideal assembly would have as many scaffolds as there are chromosomes in the respective organism (e.g., 22 autosomes and 2 sex chromosomes for humans). 2. Total number of base pairs: a higher number of total base pairs is deemed better, where the additional base pairs should be N characters that mark the space between contigs within scaffolds. 3. GC%: the ratio of G and C bases. We do not expect significant changes in G+C content; however, it may decrease slightly due to the newly inserted N characters in scaffolding (see above). 4. Assembly contiguity: we used both N50 and N90 metrics. When scaffold lengths are summed up in decreasing order, N50 corresponds to the length of the scaffold when the summation just exceeds 50% of the total assembly length. A higher number is deemed better since it shows that the assembly is less fragmented. N90 is calculated similarly, but the summation of the lengths is required to exceed 90% of the total assembly length. A higher number is deemed better.

## 3.2. Scaoflding without PCS information

We first applied scaffolding tools using the PCS dataset but without using the pool information. The results are summarized in Table 1 for chromosome 1 and in Table 2 for chromosome 20. Unfortunately, SCARPA failed in the chromosome 1 experiment due to excessive memory usage. Although BESST resulted in a lower number of scaffolds and higher N50 and N90 values for both chromosomes 1 and 20, it also returned a lower total number of base pairs. This is because BESST removed those contigs it deemed incorrectly assembled based on read mapping properties. There exist two algorithms, namely Opera and SSPACE, that can decrease resulting scaffold numbers while increasing the grand total of base pairs.

**Table 1:** Statistics of scaffolding chromosome 1 without PCS.

Tools	# Scaffolds	# Base pairs	GC%	N50	N90
Baseline	9977	121,404,443	41.57	28,272	5757
SSPACE	9891	121,405,472	41.57	28,279	5757
SCARPA*	NA	NA	NA	NA	NA
Opera	9408	121,412,030	41.57	28,159	5757
BESST	7028	99,697,046	42.12	32,938	6709

*SCARPA run failed due to excessive memory usage. ‘Baseline’ refers to the original contigs as assembled using ALLPATHS-LG.

**Table 2:** Statistics of scaffolding chromosome 20 without PCS.

Tools	# Scaffolds	# Base pairs	GC%	N50	N90
Baseline	250	10,019,750	44.75	49,769	22,871
SSPACE	249	10,019,741	44.75	49,769	22,871
SCARPA	248	10,019,787	44.75	50,183	22,871
Opera	248	10,019,760	44.75	50,183	23,331
BESST	115	4,683,891	45.44	48,117	23,589

‘Baseline’ refers to the original contigs as assembled using ALLPATHS-LG.

## 3.3. Hierarchical scaffolding using PCS

Next we repeated the scaffolding experiment using the same dataset, but this time making use of the pooling information. For this purpose, we ran scaffolding tools one pool at a time and repeated the scaffolding runs until all pools were processed. This strategy lowered the probability of using reads that originate from repeats in the same run of scaffolding.

Tables 3 and 4 summarize the scaffolding results for chromosomes 1 and 20, respectively. Once again, SCARPA failed due to high memory usage for chromosome 1, and SSPACE failed to scaffold chromosome 20. Overall, Opera yielded the best N50 and N90 values, and the BESST algorithm removed most of the data from the assembly. We observed that BESST performed worse with the pool information.

**Table 3:** Statistics of scaffolding chromosome 1 using PCS.

Tools	# Scaffolds	# Base pairs	GC%	N50	N90
Baseline	9977	121,404,443	41.57	27,634	4668
SSPACE	9569	121,501,965	41.57	29,121	5936
SCARPA*	NA	NA	NA	NA	NA
Opera	9897	121,406,580	41.57	28,531	5757
BESST	513	1,564,335	50.66	4520	1319

*SCARPA run failed due to excessive memory usage. ‘Baseline’ refers to the original contigs as assembled using ALLPATHS-LG.

**Table 4:** Statistics of scaffolding chromosome 20 using PCS.

Tools	# Scaffolds	# Base pairs	GC%	N50	N90
baseline	250	10,019,750	44.75	49,272	22,521
SSPACE*	NA	NA	NA	NA	NA
SCARPA	247	10,019,775	44.75	50,018	23,331
Opera	250	10,019,760	44.75	49,272	22,521
BESST	17	4,683,891	45.44	22,521	10,538

*SSPACE run failed. ‘Baseline’ refers to the original contigs as assembled using ALLPATHS-LG.

## 3.4. Evaluation

The hierarchical/iterative scaffolding strategy yielded slightly better results in terms of N50 and N90 statistics. We note that the sequencing depth of coverage for the PCS data was very small (3–4×), and the accuracy gain could further be improved with the availability of more sequence coverage.

We analyzed the effects of minimum number of read pairs supporting links between contigs to assembly quality. By default, the minimum number of read pairs supporting links between contigs is set to 5 in all four scaffolding algorithms. Since the PCS dataset that we used in this experiment had only about 4× coverage, we reduced this threshold to 2. However, this lowered threshold did not produce any significantly different results compared to the default value.

## 4. Discussion

The genome assembly problem is typically solved by a two-stage process: contig assembly followed by *SCARPA run failed due to excessive memory usage. ‘Baseline’ refers to the original contigs as assembled using ALLPATHS-LG. ‘Baseline’ refers to the original contigs as assembled using ALLPATHS-LG. *SSPACE run failed. ‘Baseline’ refers to the original contigs as assembled using ALLPATHS-LG. scaffolding. Obtaining longer scaffolds is of importance for achieving a more complete assembly. However, similar to the contig assembly, scaffolding is also highly prone to errors, especially when it is generated using short reads or repetitive sequences.

Even small genomes, such as those of bacteria, contain significant numbers of repeats, and it is extremely difficult, if not impossible, to assemble the human genome using short reads only (Treangen and Salzberg, 2012; Steinberg et al., 2017). De novo assembly with short reads results in a set of contigs with gaps at each repeat region that are longer than read lengths. To bridge these gaps, BAC libraries are very useful when sufficient coverage is obtained. For this reason, we decided to use a BAC library that was split into 288 pools, providing about 5% physical coverage of the genome.

Here we evaluated the performance of several commonly used state-of the-art genome scaffolds. We specifically tested whether the extra long range information obtained by PCS improved the scaffold contiguity compared to more traditional WGS-based scaffolding. We demonstrated marginal improvement in N50 and N90 statistics when the pool information was used; however, this gain in scaffolding accuracy can be improved if the depth of coverage is increased. We also observed that the scaffolds vary in their usability, speed, and accuracy. Overall, SSPACE is very useful since it is very easy to install and run. BESST is good at making joins in an aggressive way. Opera and SCARPA are better when handling misassemblies.

Although we tried to enlarge sequences into scaffolds, we recognized that resulting scaffold total base pairs are less than the total number of base pairs in the original contigs. We think that this is an important source of error of scaffolding tools. Possible reason for this might be as follows. After scaffolding processes, we expect an increment of the total number of base pairs or at least not a decrease because in the process of scaffolding contigs are sorted and gaps between different contigs are filled with N characters, N being the number of bases in the gap. The main reason for reduction in the base pair number may be the elimination of the contigs that cannot be ordered or oriented.

As a future work, it would be interesting to see the effects of the size of the pooled clones, i.e. using 40 kb fosmids versus 150 kb BAC clones. Additionally, a comparison of the more recent linked-read sequencing technologies such as 10x Genomics and Hi-C based scaffolding such as Dovetail Genomics data would be beneficial for the community.

## Acknowledgments

This study was supported by a TÜBİTAK grant (112E135) to the second author.
